# Efficacy evaluation of neuromuscular blocking agents as adjuncts to intravenous regional anesthesia: a meta-analysis of randomized controlled trials

**DOI:** 10.3389/fmed.2025.1574711

**Published:** 2025-07-11

**Authors:** Yan Yang, Shujun Sun, Guobin Song, Jianfeng Zhang, Rui Chen

**Affiliations:** ^1^Department of Anesthesiology, The First People’s Hospital of Jiangxia District, Wuhan, Hubei, China; ^2^Department of Anesthesiology, Union Hospital, Tongji Medical College, Huazhong University of Science and Technology, Wuhan, China; ^3^Department of Anesthesiology, Xiangyang Central Hospital, Affiliated Hospital of Hubei University of Arts and Science, Xiangyang, China; ^4^Department of Anesthesiology, Zhejiang Hospital, Hangzhou, China

**Keywords:** neuromuscular blocking agents, intravenous regional anesthesia, meta-analysis, randomized controlled trial, adjuncts

## Abstract

**Purpose:**

To evaluate the efficacy of neuromuscular blocking agents (NMBAs) as adjuncts to intravenous regional anesthesia (IVRA).

**Methods:**

Two researchers independently searched PUBMED, EMBASE, the Cochrane Library databases, and CBM for randomized controlled trials assessing the efficacy of NMBAs as adjuvants in IVRA.

**Results:**

This meta-analysis included 420 patients from 7 randomized controlled trials. Compared to IVRA using local anesthetics alone, the adjunctive use of NMBAs significantly shortened the onset time of sensory block [mean difference (MD) = −1.38 min, 95% CI: −2.02 to −0.75; *P* < 0.01] and motor block (MD = −2.39 min, 95% CI: −4.67 to −0.12; *P* = 0.04). Moreover, NMBAs prolonged the duration of motor block (MD = 6.97 min, 95% CI: 0.06 to 13.88; *P* = 0.05). However, no significant improvement was observed in the duration of pain relief (MD = 4.24 min, 95% CI: −1.43 to 9.91; *P* = 0.14).

**Conclusion:**

As adjuncts to IVRA, NMBAs significantly reduce the onset time of sensory and motor blocks compared to local anesthetics alone. Additionally, NMBAs prolong the duration of motor block. These agents enhance the efficacy of IVRA by optimizing neuromuscular blockade while maintaining anesthetic quality comparable to standard IVRA techniques.

## Introduction

Intravenous regional anesthesia serves as an effective alternative to peripheral nerve blocks (PNBs) or general anesthesia (GA) for upper extremity surgery. When PNBs prove insufficient, GA typically functions as the preferred rescue technique. However, in patients at high risk for GA complications, IVRA may alternatively serve as a rescue option - though its use warrants careful consideration of local anesthetic toxicity risks, particularly upon tourniquet release (1). Nevertheless, IVRA has certain limitations, such as slow onset of anesthesia, insufficient muscle relaxation, rapid recurrence of postoperative pain, and potential local anesthetic systemic toxicity (LAST) following tourniquet deflation (2). These challenges necessitate continued exploration of novel adjuvants to optimize IVRA efficacy and safety.

Neuromuscular blocking agents induce muscle relaxation and reduce spasms by modulating muscle spindle activity. Clinical studies demonstrate that adjuncts such as atracurium (2, 3), cisatracurium (4), and pancuronium (5) significantly enhance surgical conditions through improved muscle relaxation. Evidence indicates that non-depolarizing NMBAs added to local anesthetics accelerates the onset of motor block and achieves deeper muscle relaxation (3). However, these benefits may be offset by prolonged motor function recovery. This meta-analysis systematically evaluates the efficacy and clinical characteristics of NMBAs as adjuncts to local anesthetics in IVRA.

While previous research has predominantly focused on the efficacy of local anesthetics, evidence regarding the effectiveness of NMBAs in IVRA remains limited. This meta-analysis therefore specifically evaluates: (1) the impact of NMBAs on sensory block onset; (2) the onset and duration of motor block; and (3) the duration of pain relief in IVRA. Our findings provide novel insights for optimizing IVRA protocols.

## Methods

**FIGURE 1:**
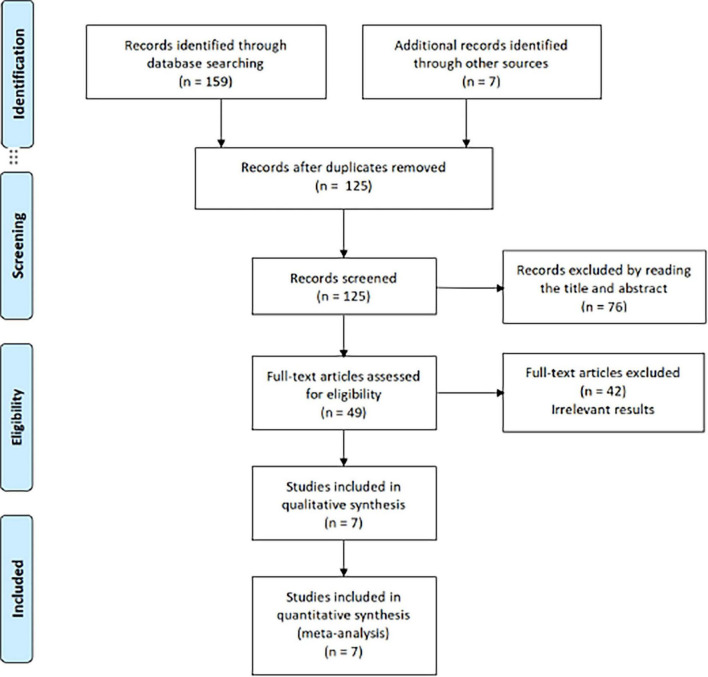
PRISMA flowchart.

This meta-analysis protocol is registered with the International Prospective Register of Systematic Reviews (PROSPERO), under registration number CRD42022363174, dated October 8, 2022. We adhered to the CONSORT 2010 checklist for reporting randomized trials, and the PRISMA flowchart is depicted in Figure 1.

## Search strategy

Two researchers independently conducted comprehensive literature searches in the PUBMED, EMBASE, CBM, and Cochrane Library databases. The search strategy involved the use of MeSH terms and keywords, including “neuromuscular blocking agents,” “intravenous regional anesthesia,” and “randomized controlled trial.” The searches were last updated on April 18, 2025. Additionally, to ensure a thorough review, the researchers also examined the reference lists of the identified articles.

## Study inclusion criteria

The original studies included were selected based on the PICOS criteria: P - patients receiving IVRA; I - the application of neuromuscular blocking agents in IVRA; C - comparison between the use of adjuvants and no adjuvants; O - primary outcomes were the onset times for motor and sensory block, with secondary outcomes including the durations of sensory and motor block, and pain-free intervals. S - Only randomized controlled trials (RCTs) were considered eligible for inclusion. Two reviewers (Y.Y. and S.S.) independently performed the literature inclusion. Disagreements between them were resolved through discussion. If consensus couldn’t be reached, a senior third researcher (J.Z.) made the final decision.

## Data extraction

### Data extraction and study characteristics

Two investigators independently conducted data extraction using standardized forms adapted from the Cochrane guidelines. The extracted information included key study characteristics such as the first author’s name, publication year, sample size, type of local anesthetic administered, total anesthetic volume, surgical procedure, and clinical application (Table 1). Outcome data (e.g., onset/duration of sensory and motor blocks) were systematically recorded to ensure methodological consistency across all included studies. This structured approach minimized bias and aligned with best practices for meta-analytic research.

**TABLE 1:** Characteristics of the included studies.

References	Application (mg)	Sample size	(mg/kg)	Total volume	Operation
Kurt et al. (18)	NS/AF0.5/AC3	11/11/11	Lid 3	40	Hand and wrist surgery
Elhakim and Sadek (2)	NS/AC2	20/20	Lid 0.5%	40	Hand surgery
Aujla et al. (5)	NS/PAN 0.5, F0.05	50/50	Lid 0.25%	40	Upper limb surgery
Sztark et al. (9)	NS/F0.05, PAN0.5	20/20	Lid 3	40	Upper limb surgery
Esmaoglu et al. (4)	NS/CIS0.5	20/20	Lid 3	40	Elective hand surgery
Alikhani et al. (8)	NS/ROC/NG	59/59/59	Rpv 0.2%	40	Forearm surgery
Mizrak et al. (12)	NS/MIV	30/30	Lid 3	40	Carpal tunnel release

NS, normal saline; AF, alfentanil; AC, atracurium; Lid, lidocaine; KR, ketorolac; Pro, procaine; PAN, pancuronium; F, fentanyl; CIS, cisatracurium; VEC, Vecuronide; ROC, rocuronium; NG, nitroglycerin; RPV, ropivacaine; MIV, mivacurium.

### Risk of bias assessment

Two reviewers independently evaluated the methodological quality of eligible studies using the Cochrane Handbook v5.0.2, resolving any disagreements through joint discussion, with a third researcher available for arbitration if needed. The assessment criteria included the generation of a random sequence, allocation concealment, blinding, handling of incomplete outcome data, selective reporting, and the presence of other biases, which were categorized as “high risk of bias,” “uncertain risk of bias,” or “low risk of bias” (6).

### Statistical analysis

Quantitative analyses of the included RCTs were conducted using Review Manager software (version 5.3, Cochrane Collaboration, Copenhagen, Denmark). Binary outcomes were assessed using the odds ratio (OR) with a 95% confidence interval (CI), while continuous outcomes were evaluated using the mean difference (MD) or standardized mean difference (SMD) with a 95% CI (6). Testing for heterogeneity of the pooled results was performed using the I-square (I^2^) test. A random-effects model was used when a large heterogeneity was presented (I^2^ > 50%); otherwise, a fixed-effect model was used in meta-analysis (7). Sensitivity analysis was employed to identify sources of heterogeneity and to mitigate its impact on outcome stability. When significant heterogeneity was detected (I^2^ > 50%), sensitivity analyses were conducted.

### Primary outcome, secondary outcome

As per the Cochrane Manual, the primary outcomes of this study were the onset times of motor and sensory blocks. The secondary outcomes included the durations of sensory and motor blocks, as well as the pain-free recovery period. Sensory block detection involved observers using standardized acupuncture techniques to evaluate sensory block every 30 s with a 22-gauge short needle. They assessed the patient’s response to the sensory cortex, ulnar nerve, median nerve, and radial nerve inside and outside the biceps brachii muscle. The onset time of sensory block was defined as the duration from administration to complete sensory block of all the mentioned skin segments. For motor block detection, participants’ motor function was evaluated by having them bend and stretch their wrists and fingers at 30-s intervals. The inability of limbs to perform any conscious movement was defined as definite motor block, and the duration of motor block was measured from administration to achieving complete motor block. Pain was assessed using a visual analog scale from the time of tourniquet swelling to the end of the surgery (8).

## Results

### Characteristics and risk of bias of eligible studies

**FIGURE 2:**
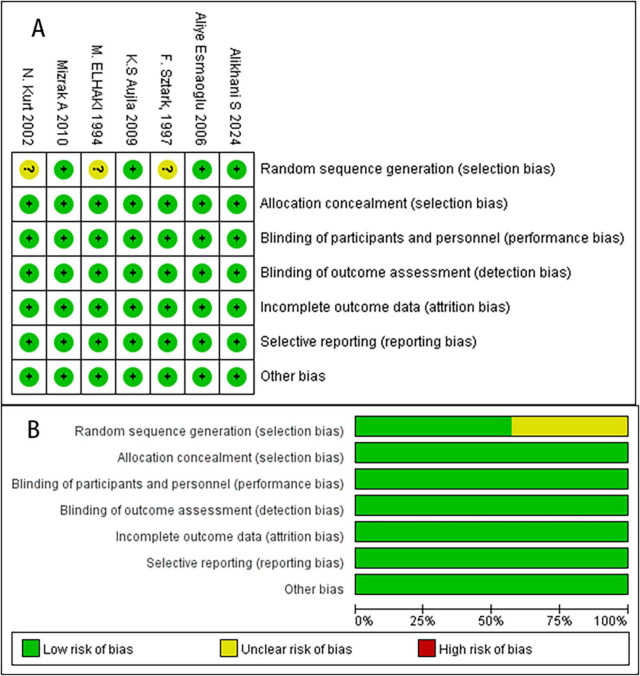
The risk of bias assessment of the included studies. **(A)** Risk bias of summary. **(B)** Risk bias of graph. There were no high risk of bias found in these studies.

The study selection process is outlined in Figure 1. We identified seven RCTs comprising 420 patients. Both reviewers demonstrated complete agreement in risk of bias assessment, indicating that the overall risk of bias in the study was low. Figure 2 presents the risk of bias summary generated using Review Manager 5.3 (RevMan). Random sequence generation employed computer-generated codes or random number tables, while allocation concealment used sequentially numbered, identical opaque envelopes prepared by an independent pharmacist. Detailed information on variable drug regimens (types and dosages) across studies is provided in Table 1.

### The onset of sensory block

**FIGURE 3:**
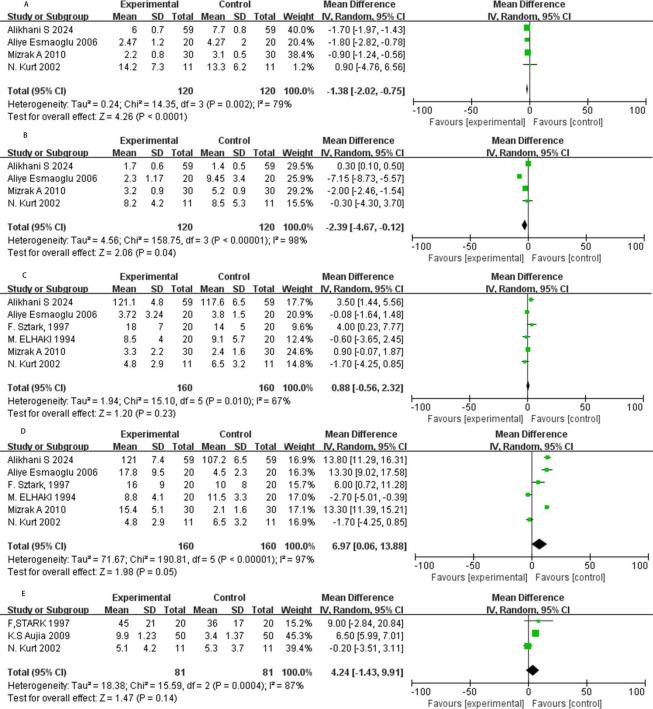
Forest plot. **(A)** The onset of sensory block in minutes. **(B)** The onset of motor block in minutes. **(C)** Duration of stable sensory block in minutes. **(D)** Duration of motor block in minutes. **(E)** Pain free period in minutes. SD, standard deviation; CI, confidence interval; IV, inverse variance.

Four RCTs reported the onset time of sensory block, and our meta-analysis adopts a random model (Figure 3A). The results showed that NMBAs significantly shorten the onset of sensory block (MD = −1.38, 95% CI: −2.02 to −0.75; *P* < 0.01, I^2^ = 79%).

### Onset of motor block

Four studies assessed the onset time of motor block with NMBAs as local anesthetics (Figure 3B). The results showed that NMBAs significantly shortened the onset of motor block (MD = −2.39, 95% CI: −4.67 to −0.12; *P* = 0.04, I^2^ = 98%).

### Duration of sensory block

Six RCTs reported the duration of stable sensory block, exhibiting moderate heterogeneity (I^2^ = 67%), prompting the use of a random model for meta-analysis (Figure 3C). The result has no statistical significance (MD = 0.88, 95% CI: −0.56 to 2.32, *P* = 0.23, I^2^ = 67%).

### Duration of motor block

Six RCTs assessed the duration of motor block, and we adopt a random model (Figure 3D). The data shows that NMBAs prolonged the duration of motor block (MD = 6.97, 95% CI: 0.06 to 13.88, *P* = 0.05, I^2^ = 97%).

### Pain-free period

Among the three studies reviewed, the meta-analysis showed that NMBAs supplements prolonged the duration of pain relief compared to the control group, but the results were not statistically significant (MD = 4.24, 95% CI: −1.43 to 9.91; *P* = 0.14, I^2^ = 87%; Figure 3E).

### Sensitivity analysis

**FIGURE 4:**
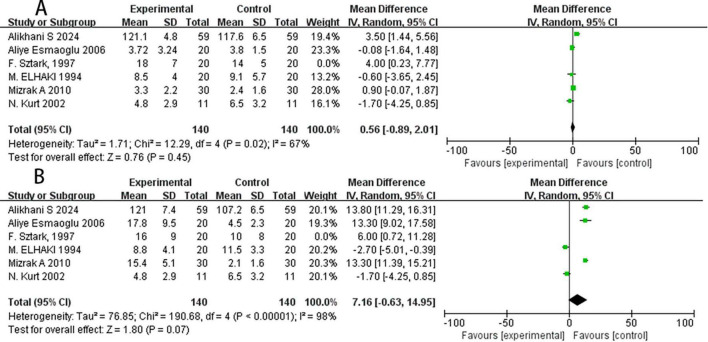
Forest plot for sensitivity analysis. **(A)** Sensitivity analysis of the duration of sensory block, **(B)** sensitivity analysis of the duration of motor block. SD, standard deviation; CI, confidence interval; IV, inverse variance.

Significant heterogeneity was observed in the above outcome metrics, and sensitivity analyses were performed to explore potential sources of this heterogeneity. Notably, Sztark et al. (9) utilized a combination of pancuronium (a NMBA) and fentanyl (an opioid) as adjuncts to local anesthetics, while the other six included studies exclusively evaluated NMBAs without opioids. We therefore excluded this study and re-analyzed the data for sensory and motor block duration using the random-effects model. The pooled estimate for sensory block duration showed no statistically significant difference between groups (MD = 0.56, 95% CI: −0.89 to 2.01, *P* = 0.45, I^2^ = 67%; Figure 4A). Similarly, the pooled estimate for motor block duration also demonstrated no statistically significant difference (MD = 7.16, 95% CI: −0.63 to 14.95, *P* = 0.07, I^2^ = 98%; Figure 4B), which contradicts the previous conclusion. Interestingly, even when we excluded Sztark et al. (9), there was no significant reduction in the inhibitory effect of the above results.

## Discussion

Intravenous regional anesthesia remains a valuable technique for upper extremity surgery, particularly involving the hand and wrist, offering simplicity, reliability, and cost-effectiveness, especially for short-duration procedures (10). Nevertheless, inherent limitations persist, including concerns over potential LAST, a relatively slow onset of anesthesia, insufficient muscle relaxation at the surgical site, and rapid recurrence of postoperative pain following tourniquet deflation (1). To address these shortcomings, particularly the need for accelerated onset and improved intraoperative conditions, various adjuncts have been investigated for use with local anesthetics in IVRA. Among these are NMBAs such as atracurium (2), mivacurium (11, 12), pancuronium (13), cisatracurium (4), vecuronium (14), and rocuronium (8).

The primary mechanism of NMBAs involves competitive antagonism of nicotinic acetylcholine receptors at the neuromuscular junction. This action prevents depolarization of the motor endplate, leading to skeletal muscle relaxation (15). Crucially, this mechanism targets motor nerve transmission and has minimal direct effect on sensory nerve function or nociception, which are primarily modulated by local anesthetics acting via sodium channel blockade in neuronal axons (16). Consequently, the rationale for NMBA use in IVRA centers on enhancing motor block and surgical conditions rather than directly influencing sensory blockade or analgesia.

Our meta-analysis, synthesizing data from 7 randomized controlled trials, provides evidence supporting this rationale. We found that adjunctive NMBAs significantly shortened the onset time of both sensory (MD = −1.38 min) and motor blocks (MD = −2.39 min) compared to local anesthetic alone. Furthermore, NMBAs prolonged the duration of motor block (MD = 6.97 min). However, consistent with their mechanism of action, NMBAs did not significantly prolong the duration of sensory block or the pain-free period. These findings align with the established pharmacology: NMBAs primarily enhance motor effects. The observed acceleration in sensory block onset, while statistically significant, is modest and its clinical relevance may be limited; it could potentially stem indirectly from improved local anesthetic distribution facilitated by muscle relaxation or reduced movement artifact during assessment, rather than a direct pharmacodynamic effect on sensory nerves. This interpretation is supported by studies such as Esmaoglu et al. (4) demonstrating accelerated sensory and motor onset with cisatracurium, and Mizrak et al. (12) confirming faster motor onset with mivacurium.

The benefit of enhanced motor relaxation must be balanced against the potential for prolonged motor recovery, as evidenced by our finding of increased motor block duration. While this prolongation meets surgical demands for relaxation, it necessitates awareness of the extended time until full motor function returns after tourniquet release, possibly influenced by residual receptor blockade (15). Although theoretically, muscle spindle blockade by NMBAs could alleviate spasms and potentially reduce pain (17), our meta-analysis found no significant evidence supporting improved intraoperative or postoperative analgesia (pain-free period).

Notably, significant heterogeneity (I^2^ > 50%) was observed across several outcomes, including sensory and motor block onset and duration. This heterogeneity likely originates from several sources: diversity in patient populations (surgical patients vs. healthy volunteers), variations in the specific NMBA used and its dosage, differences in the type and concentration of the primary local anesthetic (e.g., lidocaine vs. procaine), inconsistencies in the methods used to assess block onset and duration, and variations in surgical procedures. Sensitivity analyses were conducted to explore this heterogeneity. For instance, excluding the study by Sztark et al. (9), which uniquely combined an NMBA (pancuronium) with an opioid (fentanyl), altered the statistical significance for motor block duration, highlighting how differing adjunct combinations contribute to variability. Choosing a fixed-effects model over a random-effects model for some analyses also impacted significance, further emphasizing the need for cautious interpretation.

Safety considerations are paramount when using NMBAs. The primary concern is their potential to cause respiratory depression, typically associated with systemic absorption in general anesthesia. However, studies within the IVRA context suggest that low doses of NMBAs, confined predominantly to the isolated limb, result in fewer systemic side effects, with transient diplopia being occasionally reported (3). Research indicates that adding modest amounts of NMBAs to the IVRA mixture can achieve satisfactory muscle relaxation for upper limb surgery without significant adverse effects in both volunteers and patients (e.g., wrist fractures) (13). Nevertheless, vigilance for potential systemic effects, particularly upon tourniquet release, remains essential.

Our study has limitations that warrant consideration. Firstly, restricting the literature search to English and Chinese databases may have omitted relevant studies published in other languages. Secondly, the substantial heterogeneity observed across studies complicates the drawing of definitive conclusions and underscores the need for standardized protocols in future research regarding drug regimens, dosages, and outcome assessments. Thirdly, the relatively small number of included trials (*n* = 7) and patients (*n* = 420) limits the statistical power, particularly for secondary outcomes like the pain-free period. Finally, the sensitivity of results to the choice of statistical model (fixed vs. random effects) and the exclusion of specific studies highlights the fragility of some findings and the need for larger, more homogeneous trials.

In conclusion, this meta-analysis demonstrates that neuromuscular blocking agents, when used as adjuncts to local anesthetics in IVRA, effectively accelerate the onset of both sensory and motor blockade and prolong the duration of motor block. These benefits optimize surgical conditions by enhancing muscle relaxation. While NMBAs do not significantly extend the duration of analgesia, their use may allow for a reduction in the required dose of local anesthetic, potentially mitigating the risk of local anesthetic systemic toxicity. Adjunctive NMBAs represent a valuable option for IVRA procedures where improved intraoperative muscle relaxation is a primary objective. Future research employing standardized methodologies and larger sample sizes is needed to further refine their role and confirm safety profiles.
